# A Study to Investigate the Prevalence of Device-Specific Errors in Inhaler Technique in Adults With Airway Disease (The SCORES Study): Protocol for a Single Visit Prevalence Study

**DOI:** 10.2196/26350

**Published:** 2021-08-27

**Authors:** Ruth De Vos, Thomas Brown, Jayne Longstaff, Mitch Lomax, Heather Mackenzie, Alexander Hicks, Hitasha Rupani, Jessica Gates, Lauren Fox, Laura Wiffen, Anoop J Chauhan

**Affiliations:** 1 Portsmouth Hospitals University NHS Trust Research and Innovation Department Portsmouth United Kingdom; 2 School of Sport, Health and Applied Physiology University of Portsmouth Portsmouth United Kingdom; 3 University of Southampton Southampton United Kingdom; 4 Faculty of Science and Health University of Portsmouth Portsmouth United Kingdom

**Keywords:** inhaler, inhaler technique, inhaler technique error, asthma, COPD

## Abstract

**Background:**

It is a recurring theme in clinical practice that patients using inhaled medications via an inhaler do not use their device to a standard that allows for optimum therapeutic effect, and some studies have shown that up to 90% of people do not use their inhalers properly. Observation and correction of the inhaler technique by health care professionals is advised by both national and international guidelines and should be performed at every opportunity to ensure that the optimum inhaler technique is achieved by the user. This study will provide a greater understanding of the most frequent technique errors made by people using 13 different inhaler types.

**Objective:**

This study aims to identify and compare inhaler technique errors and their prevalence in adults, using device-specific checklists in accordance with manufacturers’ guidelines, for 13 specific inhaler types across all lung conditions and to correlate these errors with possible determinants of poor technique. It also aims to assess the error frequency at each step in the device-specific questionnaires and compare the error rates among device types.

**Methods:**

In a single visit, participants using an inhaler included in the inclusion criteria will have their inhaler technique observed using an identical placebo device, which will be recorded using device-specific checklists, and technique-optimized, or switched to a suitable inhaler.

**Results:**

The study is already underway, and it is anticipated that the results will be available by 2022.

**Conclusions:**

The SCORES (Study to Investigate the Prevalence of Device-Specific Errors in Inhaler Technique in Adults With Airway Disease) study will ascertain the prevalence of device-specific inhaler technique errors at each step in the device-specific checklists, compare error rates among 13 device types, and correlate these errors with possible determinants of poor technique. Future work will involve the clarification and classification of these errors into *critical* and *noncritical* categories.

**Trial Registration:**

ClinicalTrials.gov NCT04262271; https://clinicaltrials.gov/ct2/show/NCT04262271

**International Registered Report Identifier (IRRID):**

DERR1-10.2196/26350

## Introduction

### Background

Inhaled medications are the cornerstone of pharmacological treatment for many lung conditions, such as asthma and chronic obstructive pulmonary disease (COPD), and the amount spent by the National Health Service (NHS) on inhaled medication is considerable [1], with poor inhaler technique accounting for additional indirect costs [2].

It is a recurring theme in clinical practice that patients with asthma and COPD alike do not use their inhalers to a standard that allows optimum therapeutic effect [3-7]. Many patients continue to make critical errors when using their inhalers; therefore, they do not realize or enjoy the benefits of optimal inhaled treatment. Consequently, the NHS spends millions of pounds on high-cost yet subtherapeutic treatment [8]. In addition, there is now a burgeoning number of inhaler devices available for patient use, which causes additional confusion among health care professionals on how to reliably evaluate and quantify a good inhaler technique to a standard that allows quality assurance [9,10].

Both the Global Initiative for Asthma (GINA) [11] and Global Initiative for Chronic Obstructive Lung Disease (GOLD) [12] place great importance on correcting poor inhaler technique before escalating inhaled therapy. There is also a large body of evidence reporting that inhalation errors are associated with worse disease outcomes in patients with asthma and COPD and that the time invested by health care professionals to correct patients’ inhaler technique is vital to improve health outcomes [13].

Other than device-specific checklists based on manufacturer’s guidelines, which are often not used or shared in any standardized fashion, there is currently no formal way of assessing and quantifying the inhaler technique errors (ITE) made by patients in a way that can be translated between health care environments. Divergence and heterogeneity between checklists for the same device in previously published studies makes direct comparison of results difficult; thus, development of common checklists has been recommended [14].

### Objectives

#### Overview

This study aims to identify ITE and their prevalence using device-specific checklists based on manufacturers’ guidelines for 13 inhaler types across all lung conditions and to correlate these errors with possible determinants of poor technique. It also aims to assess the error frequency at each step in the device-specific questionnaires, compare error rates among device types, and determine which of these errors are deemed *critical* or *noncritical*.

#### Primary Objective

The primary objective of this study is to assess the prevalence of device-specific ITE in 13 different devices, using the manufacturers’ guidelines.

#### Secondary Objectives

The secondary objectives are as follows:

1. To assess whether the frequency of ITE are associated with participant demographics, respiratory diagnosis, and comorbidities; disease control as measured by questionnaires, such as Asthma Control Questionnaire (ACQ) for asthma and COPD Assessment Tool (CAT) score for COPD, and participant clinical characteristics including exacerbation frequency within the past 12 months; length of time a participant has been using the observed inhaler, along with previous device-specific inhaler technique training (if and when previous training has taken place and given by whom); and levels of behavioral and adherence risk profile as measured by the social, psychographic, usage, and rational (SPUR) profiling tool (Observia).
2. To assess the user error frequency at each step in the device-specific guidelines.
3. To compare user error rates among device types.

#### Exploratory Objectives

The exploratory objectives are as follows:

1. To categorize ITE as *critical* or *noncritical* and prioritize the importance of these errors by conducting a further study outside the remit of this protocol and involves a Delphi consensus process. Owing to ongoing debate and variations in the definitions of critical and noncritical errors, final definitions will be based on the outcomes of the Delphi consensus process. The information from the Delphi process, along with the data from this prevalence study, will be collated to determine which ITE will be included in a subsequent study, in which a scoring system to quantify ITE will be developed.
2. To correlate severity of disease with ITE if a disease-specific tool is available. The GINA step will be used for asthma and GOLD stage for COPD with spirometry, whereas forced expiratory volume in 1 second (FEV_1_), forced vital capacity (FVC), and FEV_1_/FVC ratio measurements will be used if spirometry is performed as part of standard care.

## Methods

### Overview

This is a prevalence study and includes both descriptive and analytical methods, analyzing the prevalence of ITE in adults with airway disease across 13 different inhaled devices.

### Eligibility Criteria

Participants will be drawn from a range of clinical conditions affecting adults that use an inhaler device. The inclusion and exclusion criteria are presented in Textbox 1.

**Inclusion and exclusion criteria.**

**Inclusion criteria**

- Participants must be aged ≥18 years.
- Participants should have been prescribed (by a doctor or health care professional) 1 of 13 inhaler device types for an airways condition (Accuhaler, Autohaler, Breezhaler, Easi-breathe, Easyhaler, Ellipta, Genuair, Handihaler, Nexthaler, pressurized metered-dose inhalers, pressurized metered-dose inhalers plus spacer [eg, Aerochamber or Volumatic], Respimat, and Turbohaler).
- Participants should be able to provide written informed consent.

**Exclusion criteria**

- Participants are currently on treatment with systemic steroids or antibiotics for an exacerbation of the participants’ airway condition.
- In the opinion of the investigator, the participant will be unable to perform the study procedures.

### Sampling and Sample Size

A minimum of 650 participants will be recruited for the study. To ensure an adequate sample size for each of the 13 different device types, a minimum of 50 participants will be recruited for each device group. Owing to the wide variety of devices involved in the study, it was not possible to conduct a power calculation because the device error frequency is known to be disparate among the inhalers involved. On the basis of existing recruitment numbers in published ITE trials [15], alongside the expert clinical opinion from within the protocol development team, a minimum of 50 participants using each device type was felt appropriate to provide a reasonable chance to identify a range of errors.

Potentially, participants may be using several different inhaler device types, so there could be a choice of device type to observe. Throughout the study, the study team will closely monitor the number of participants recruited using each of the 13 device types; therefore, to ensure that the risk of selection bias is distributed equally across all device types, the least commonly recruited device type will be observed in each participant.

Careful consideration has been afforded to ensure that adequate numbers of participants are recruited in all 13 device types included in the study. We will have access to more than 70,000 patients across the Wessex region using participant identification center ITE. Examination of primary care databases will identify those using the inhaler types included in this study, and invitations to participate will be sent to ensure recruitment across all device types.

Recruitment will last for up to 24 months and will not cease at the recruitment of 50 participants in each group, allowing sample sizes greater than this figure.

### Recruitment

Participant recruitment into the study, undertaken by members of the research study team who are also part of the respiratory clinical care team at Queen Alexandra Hospital (Portsmouth Hospital University NHS Trust), will be via outpatient clinics, inpatient wards, research clinics in primary care, respiratory support groups, general practitioner (GP) practices as participant identification center ITE, social media feeds (Facebook and Twitter) of Portsmouth University Hospitals, staff and volunteers within the Trust, posters and flyers throughout the hospital, and the research database (in accordance with General Data Protection Regulation) compiled by the research department, which includes participants from previous research studies who have consented to be contacted for future studies.

Participants will be issued with a participant information sheet and will have adequate time to read this information before enrollment. Written consent will be obtained before undertaking any study-related activities.

Potential participants approached within clinical settings, who do not then consent to participate in the study, will be assured that nonparticipation will not affect their ongoing medical treatment, that their inhaler technique will be observed as per normal practice, and that they will be reeducated as necessary.

### Study Assessments

This section describes the information that will be recorded in the participant’s case report form (CRF), which will be anonymized using the participant’s unique study number.

#### Participant Clinical Characteristics

This includes patient demographics, such as age and gender, diagnosis requiring inhaler use, and comorbidities.

#### Disease Control Questionnaires

Disease control will be assessed using the ACQ for participants with asthma and the CAT for participants with COPD. For participants with a respiratory diagnosis other than asthma or COPD, this will be assessed by recording the number of exacerbations of their respiratory condition in the previous 12 months. An exacerbation is defined as an acute worsening of symptoms requiring treatment with antibiotics or oral corticosteroids. The ACQ [16] is a validated questionnaire for assessing the level of asthma control over the preceding 7 days. The ACQ-6 will be recorded if spirometry is not recorded for the purpose of the study, otherwise the ACQ-7 will be used. The COPD Assessment Test [17] is a validated, 8-item unidimensional measure of health status impairment in COPD. It assists patients and their physicians in quantifying the impact of COPD on the patient’s health. Responses are given on a 5-point scale, with a maximum total score of 40. The higher the score, the greater the impact COPD has on the patient’s health; scores of 0-9 are considered low impact; 10-20, medium; 21-30, high; and >30, very high.

#### Disease Severity

If possible, disease severity will be categorized. For participants with asthma, it will be step 1-5, as per the GINA guidelines. In patients with COPD, this will be categorized as A-D using the GOLD guidelines with the severity of airflow limitation based on current or last available spirometry results within the last 12 months combined with their CAT score and exacerbation history. For participants with respiratory conditions other than asthma and COPD, the number of exacerbations in the last 12 months and lung function, if available, will be used to determine disease severity.

#### Spirometry

Spirometry will be conducted using a spirometer conforming to American Thoracic Society/European Respiratory Society standards, as specified by the manufacturer’s instructions. Participants will inhale rapidly and completely from functional residual capacity, then exhale in an initial forced exhalation and continue exhalation until the end of the breath. FEV_1_ (L), FVC (L), and FEV_1_/FVC ratio will be recorded. FEV_1_ and FVC will be documented as both absolute values and as a percentage of the predicted value [18]. A minimum of 3 tests will be performed, with 2 tests within 100 mL or 5% of each other. Spirometry results taken within the last 12 months will be recorded if the current results are unavailable. Spirometry will only be performed and recorded in the CRF if it is required as part of the participant’s routine care standard care (eg, as part of a clinical appointment). For participants recruited outside of the clinical setting, spirometry will not be performed as part of the study assessment.

#### Previous Inhaler Technique Training

The date of the participants’ most recent inhaler technique assessment for the device being observed during the study visit will be recorded in the CRF, along with the cadre of the person by whom it was assessed (eg, nurse, pharmacist, physiotherapist, or doctor). We will also record which inhaler device the participant is observed using and how long they have been using this device for.

#### SPUR Profiling Tool

The SPUR profiling tool summarizes the understanding of patient behavior and their health beliefs into four drivers: social (ie, support from family and society), psychographic (ie, beliefs on identity and reactance to authority), usage (ie, financial or personal barriers), and rational (ie, decisions based on burdens to overcome and the gravity of their condition). It has been developed by the company Observia and is a comprehensive way of assessing an individual’s level of behavioral and adherence profile. The tool is designed to be dynamic, so participants progress through the questions based on the responses to previous questions, which takes approximately 7 minutes to complete and is completed using an iPad, tablet, or smart phone with a user-friendly interface. Participants will be asked up to 17 questions, and the SPUR output will be calculated. The first 4 questions are not directly related to the participants’ situation but are hypothetical situations of other patients, which is called the Vignette technique [19]. Responding to the circumstances of a third party (ie, other patients) not only allows for stronger reactions in a less threatening way but also elicits starting assumptions as a primer for the rest of the assessments. The results of the SPUR profile will not be disclosed to the patient and only be recorded in the CRF and stored by Observia, as this study does not intend to inform patient management decisions.

The data collected will be anonymized with the participants’ unique study number and will only be collected for the purpose of the study. This profiling tool will be used for each participant in the presence of a member of the research team. Observia will host the data collected during the study by Avenir Télématique, an accredited health care data hoster based in France. Observia and Avenir Télématique both act in compliance with the General Data Protection Regulation requirements.

#### Device-Specific Error Frequency

Each of the device-specific checklists, devised from manufacturers’ guidelines, includes steps to follow as recommended by manufacturers to achieve a good inhaler technique. Each step carried out by the participant will be recorded as a dichotomous yes or no answer. There will also be space on the checklist to record additional errors that may not have been recognized.

#### Error Rates

The error rates recorded for each device type will be compared with errors made in other devices in the statistical analysis.

### Inhaler Technique Observation and Intervention

Once consent has been obtained, participants will be invited to a suitable clinic room to ensure privacy and a controlled environment with no time pressure. They will be accompanied by a member of the study research team, and the same person will both assess and correct any identified errors in the inhaler technique. The study team members will also record all relevant study data into the CRF. The participants will be asked to demonstrate their inhaler technique using a single-use placebo inhaler device. This is standard clinical practice, and placebo devices, which are identical to the inhaler, are available for all inhaler device types. The technique for using a placebo device is identical to how they would use their inhaler.

The member of the study team will first observe the participant’s inhaler technique using the placebo device for the inhaler that the participant has been prescribed. Any errors in the inhaler technique will be recorded against the device-specific checklist formulated using the manufacturer’s guidelines. This checklist, unique to each inhaler device, includes specific recommended steps to be followed to achieve an optimum inhaler technique, along with any errors in the technique that have been observed.

Participants who are observed to make errors with their inhaler technique will be given feedback by the study team member, and any errors will be highlighted and discussed. The correct technique will be demonstrated by the study team member, with emphasis on correction of the errors, and the participant will then be asked to repeat the demonstration of the inhaler technique to ensure that the errors have been corrected. This method of inhaler technique correction is part of standard clinical practice. A written handout with the correct technique for that device will also be issued to the participant, so that they can refer to this to continue to implement the correct technique.

If a participant is observed making repeated errors using their device, despite inhaler technique correction, a more suitable inhaler device will be considered. If it is possible to obtain a prescription from a qualified health care professional for an alternative, more suitable device, then this device will be issued during the study visit. The participant will be taught how to use the new device and will be asked to demonstrate the correct technique as per standard clinical practice. Observations with ITE will be recorded in the participants’ CRF for the initial device only. A letter will be sent to the participants’ GP, informing them that a new inhaler device has been issued, with a request to add this to their future repeat prescriptions. If it is not possible to issue an alternative device, a letter will be sent to the participants’ GP recommending a change to a more suitable device, and the participant will require training in the use of this new device. If a participant is participating in another research trial, they will be encouraged to communicate any change in device type or medication with the investigators of the other trial.

If a participant has been prescribed and uses more than 1 inhaler device type, observation and correction of only one device type will be carried out.

To ensure that each member of the study team involved in the inhaler technique observation has the necessary expertise and to ensure standardization across device technique assessment, all members of the study team will attend regular training sessions that will continue every 3 months throughout the study to ensure that competencies have been maintained. Study investigators will have to demonstrate the perfect inhaler technique for each device to be deemed competent. A standard operating procedure for the correct inhaler technique for each device will be produced, and records of competencies will be kept in the study site file.

### Discontinuation or Withdrawal of Participants From the Study

Participants will be assured that they can withdraw from the study at any time and that they do not have to give reasons for their withdrawal. Participants who do not wish to have their inhaler technique observed and corrected will not be recruited into the study. Potential participants approached within the clinical setting of Queen Alexandra Hospital (Portsmouth), but who do not wish to be recruited into the study, will have their inhaler technique observed and will be reeducated as necessary as per normal clinical practice. They will be assured that withdrawing from the study will not affect their ongoing medical care in any way.

### Procedures for Data Management

The observation and correction of inhaler technique is part of normal clinical practice, and enrollment in the study will not be documented in the participant’s medical notes. Completed CRFs will be stored in a secure location at Queen Alexandra Hospital and can be accessed only by the research staff. All data will be recorded on paper CRFs.

The SPUR profiling tool will be administered using an iPad, tablet, or smart phone. The anonymized data will be hosted by Avenir Télématique, an accredited health care data hoster based in France. The data collected will only be collected for the purpose of this study. Observia and Avenir Télématique both act in compliance with the General Data Protection Regulation requirements.

### Data Management

A bespoke database will be created for this study. Data will be entered, and 10% of records will subsequently be randomly checked against the original CRF. Further verification will be performed according to the frequency and pattern of the errors. The research team and the Portsmouth Technology Trials Unit will carry out all data verification.

### Data Analysis

#### Overview

Errors made at each step in each specific device will be tabulated, so that errors and error frequencies can be recorded for each inhaler device against predefined checklists based on the manufacturers’ guidelines. As a minimum of 50 participants are to be recruited for each inhaler device type, there may be a difference in the number of participants in each inhaler group. To reduce bias of over- or underselection, analysis will be restricted to 50 participants using each inhaler device, and participants will be randomly selected for the analysis.

#### Summary Statistics

Summary statistics will be presented for all the background characteristics. The proportion of participants for all background variables will be presented. The information will be available as described in the following sections.

#### Primary Analysis

The primary analysis will comprise a comparison of the number of errors made for each inhaler type. As the number of errors is unlikely to be normally distributed, both mean and median values will be presented in all cases. One-way analysis of variance (ANOVA) will be used to determine whether there is a significant difference between the number of errors per inhaler type. If this parametric test is inappropriate based on the distribution of the number of errors, the Kruskal–Wallis (ranked ANOVA) will be used instead.

#### Secondary Analysis

The secondary analysis will compare the error rates for different background measures. In all cases, the choice of test will depend on whether the number of events is normally distributed. Parametric tests will be used in the first instance, unless there is evidence of nonnormality as assessed using a Shapiro–Wilk test, in which case a nonparametric alternative will be used.

CAT scores will be categorized for the purposes of analysis, with scores of 0-10, 11-20, 21-30, and 31-40 representing mild, moderate, severe, and very severe clinical impact, respectively. One-way ANOVA or Kruskal–Wallis tests will be used to assess whether there is any significant difference in the number of errors among CAT categories.

Exacerbation frequency within the last 12 months will similarly be categorized for the purposes of analysis, with the categories of xx-xx and yy-yy used. One-way ANOVA or Kruskal–Wallis tests will be used to assess whether there is any significant difference in the number of errors between CAT categories.

The association between the number of errors and continuous measures of FEV_1_, FVC, and FEV_1_/FVC ratio will be assessed using bivariate correlation. If the multivariate normality assumptions of the Pearson correlation are not met, logged values of the number of errors will be used to correct for this. If the logged values are still nonnormal, Spearman rank correlation coefficients will be used instead.

The severity of asthma will be measured using the GINA stepwise treatment guide. This measure will be categorized according to the GINA level, and comparisons between the number of errors will be assessed using a one-way ANOVA or Kruskal–Wallis test if the parametric assumptions of the former test are not met.

The severity of COPD will be categorized according to the GOLD stage, which is supplied in the form of categories. One-way ANOVA or Kruskal–Wallis tests will be used to assess whether there is any significant difference in the number of errors among GOLD levels.

Previous device training will be categorized on the basis of whether patients had received previous training. This will be classified as a binary yes or no contrast. Differences in the number of errors will be tested using an unpaired *t* test, or in the event of nonnormality, a Mann–Whitney *U* test.

Among the participants who had received previous inhaler technique training, the cadre of the person giving the training will be categorized as xx, yy, and zz. Differences in the number of errors will be tested using a one-way ANOVA test or a Kruskal–Wallis test if there is evidence of nonnormality.

#### Procedure for Missing, Unused, or Spurious Data

There are no plans for multiple imputation or other corrections for missing data.

### Ethical Considerations

#### Statement of Compliance

All staff working on this study will hold evidence of good clinical practice training before undertaking any responsibilities, and all staff working within the research study team are also part of the respiratory clinical care team. Written informed consent will be obtained from all participants after an adequate explanation of the aims, methods, and anticipated benefit of the study using the participant information sheet. A signed copy of the consent form will be given to the participant, and copies will be filed in the study master file and the participants’ medical notes.

Participants’ anonymity will be maintained throughout by identification on a password-protected electronic database and CRFs only by initials and participant ID number. All documents will be stored securely and only accessible by study staff and authorized personnel; the study will comply with the Data Protection Act.

#### Potential Benefits or Risks of Study Participation

By participating in this study, participants who are observed making errors with their inhaler technique will have these errors highlighted and will be reeducated. This is a beneficial outcome for participants’ self-care and management and ensures that they maximize drug delivery from their inhaler.

This study analyzes a wide variety of inhaler devices and will help provide a clear picture of the inhalers that are associated with the most errors. Understanding this information is important in the context of managing lung conditions, from treating patients in primary care through to feeding this information back to device manufacturers.

Any risks to the participants have been carefully considered. Participants found unable to use the inhaler device they have been prescribed will have an alternative device issued if possible, or a letter will be written to their GP with the recommended new device as per clinical practice.

#### Other Ethical Considerations

The study was approved by the Hampshire A Research Ethics Committee (REC; REC reference 19/SC/0286) in August 2019. They reviewed and approved the protocol and all relevant study materials. Any changes to the protocol or relevant study documents will be approved by the sponsor. If an amendment is made that requires REC approval, as defined by REC as a substantial amendment, the changes will not be instituted until the amendment has been reviewed and received approval or favorable opinion from the REC and research and development departments. A protocol amendment intended to eliminate an apparent immediate hazard to participants may be implemented immediately, provided that the REC is notified as soon as possible and that an approval is requested. Minor amendments as defined by REC as a nonsubstantial amendment may be implemented immediately and the REC will be informed. All participants will have adequate time to consider participation in the study, as per the Good Clinical Practice guidelines.

Patients who are already enrolled in other research trials will be invited and allowed to participate in the study if they wish. This was discussed with our Patient Public Involvement representatives who felt that these patients should have the opportunity to participate in this study and should not be excluded.

### Patient Public Involvement Process

Patient involvement in this study has been sought from patients with first-hand experience of living with chronic respiratory disease. Through face-to-face meetings, e-mail, and telephone contact, we have discussed the concept, impact, and details of the study with our respiratory patient representatives from local British Lung Foundation groups. These people have lived with respiratory conditions and have been involved in previous research studies. They contributed to developing the key questions and setting our study objectives, ensuring that we answer the questions that are relevant to people suffering from airway diseases. Assistance was also sought with participant recruitment design and the implementation of the study within a standard clinical visit to minimize delays for patients who agreed to participate in the study. They also helped design the participant information sheet and coauthored the lay summary.

## Results

Recruitment into the study has already commenced, with the study scheduled to be closed to recruitment by December 2021. It is anticipated that the results will be available by late 2022.

## Discussion

The SCORES (Study to Investigate the Prevalence of Device-Specific Errors in Inhaler Technique in Adults With Airway Disease) study will provide valuable information on the frequency of ITE. These errors will enhance health care professionals’ knowledge on this important subject, and by correcting their inhaler technique, participants will benefit from the study.

## Abbreviations

ACQ: Asthma Control Questionnaire
ANOVA: analysis of variance
CAT: Chronic Obstructive Pulmonary Disease Assessment Test
COPD: chronic obstructive pulmonary disease
CRF: case report form
FEV_1_: forced expiratory volume in 1 second
FVC: forced vital capacity
GINA: Global Initiative for Asthma
GOLD: Global Initiative for Chronic Obstructive Lung Disease
GP: general practitioner
ITE: inhaler technique errors
NHS: National Health Service
REC: Research Ethics Committee
SCORES: Study to Investigate the Prevalence of Device-Specific Errors in Inhaler Technique in Adults With Airway Disease
SPUR: social, psychographic, usage, and rational
